# An Italian Disease-Based Registry of Axial and Peripheral Spondyloarthritis: The SIRENA Study

**DOI:** 10.3389/fmed.2021.711875

**Published:** 2021-09-22

**Authors:** Alen Zabotti, Michele Maria Luchetti, Carlo Francesco Selmi, Roberta Ramonda, Rosa Daniela Grembiale, Lorenzo Dagna, Salvatore D'Angelo, Giacomo Cafaro, Salvatore De Vita, Mara Felicetti, Silvia Marelli, Daniela Frigerio, Ennio Giulio Favalli

**Affiliations:** ^1^Rheumatology Clinic, Department of Medical Area, University of Udine, Academic Hospital S. Maria della Misericordia, Udine, Italy; ^2^Clinica Medica, Dipartimento Scienze Cliniche e Molecolari, Università Politecnica delle Marche, Ancona, Italy; ^3^Rheumatology and Clinical Immunology, Humanitas Research Hospital, Humanitas University, Milan, Italy; ^4^Rheumatology Unit, Department of Medicine-DIMED, University of Padova, Padova, Italy; ^5^Rheumatology Research Unit, Dipartimento di Scienze della Salute, Università “Magna Graecia”, Catanzaro, Italy; ^6^Unit of Immunology, Rheumatology, Allergy, and Rare Diseases, IRCCS San Raffaele Scientific Institute, Milan and Vita-Salute San Raffaele University, Milan, Italy; ^7^Rheumatology Department of Lucania, Rheumatology Institute of Lucania, San Carlo Hospital, Potenza, Italy; ^8^Reumatologia, Dipartimento di Medicina, Università di Perugia, Perugia, Italy; ^9^Medical Affairs Department, Immunology, Janssen-Cilag SpA, Milan, Italy; ^10^S.C. Reumatologia ASST Centro Specialistico Ortopedico Traumatologico Gaetano Pini – CTO, Milan, Italy

**Keywords:** spondyloarthritis, peripheral spondyloarthritis, axial spondyloarthritis, psoriatic arthritis, prospective study, registry, real-world evidence

## Abstract

**Introduction:** Data about the clinical presentation and management of early and mild spondyloarthritis (SpA) are limited.

**Objectives:** The objective of this study was to describe the baseline characteristics of disease-modifying antirheumatic drug (DMARD)-naïve patients with axial or peripheral SpA.

**Methods:** The Spondyloarthritis Italian Registry: Evidence from a National Pathway (SIRENA) study is an ongoing, Italian, multicenter, prospective registry of patients with a first or newly confirmed diagnosis of SpA according to the Assessment of SpondyloArthritis International Society (ASAS) criteria. To be included, patients had to be naïve to conventional, targeted, and biological DMARDs for SpA. Patients were enrolled between June 2017 and June 2019 and classified into groups according to disease presentation: predominantly axial or peripheral manifestations. The study is ongoing, and patients are being followed for 2 years, with an evaluation every 6 months according to clinical practice. Differences in baseline demographics, lifestyle, and clinical characteristics between axial and peripheral SpA were evaluated.

**Results:** In this study, 350 patients were enrolled, of which 123 (35.1%) were axial and 227 (64.9%) were peripheral patients. Patients with axial SpA were significantly younger at enrollment (median age: 44 vs. 53 years), had significantly more anxiety/depression (13 vs. 2.6%), and expressed higher disease activity compared to patients with peripheral SpA. Patients with peripheral SpA had significantly more cardiometabolic disorders (33 vs. 18.7%), skin psoriasis (65.2 vs. 21.1%), and nail psoriasis (35.5 vs. 17.1%) than patients with axial SpA. Dactylitis, enthesitis, and fibromyalgia were observed, respectively, in 17.6, 51.2, and 5.7% of patients with axial SpA and 24.3, 40, and 3.1% of patients with peripheral SpA. In both disease groups, women tended to report depression, joint tenderness, and higher disease activity more frequently than their male counterparts. At inclusion, a new diagnosis of SpA was performed in 58% of axial and 77% of peripheral patients, with a median time from symptom onset to diagnosis of 36 and 24 months, respectively. At baseline, most patients with axial SpA (77%) started a biological DMARD, while over half of the peripheral patients started a conventional DMARD.

**Conclusions:** Based on a well-characterized clinical registry of SpA, we provided real-world insights on the clinical features of DMARD-naïve SpA patients, pointing out major differences between axial and peripheral disease in terms of clinical characteristics and treatment pattern. Future prospective evaluations within the SIRENA study will improve knowledge on SpA and contribute to defining the best therapeutic approach.

## Introduction

Spondyloarthritis (SpA) is a heterogeneous group of interrelated but phenotypically distinct rheumatic inflammatory disorders that comprise ankylosing spondylitis (AS), non-radiographic axial SpA, psoriatic arthritis (PsA), reactive arthritis (ReA), arthritis associated with inflammatory bowel disease (IBD), and undifferentiated SpA. These conditions share common clinical features, including enthesitis, dactylitis, inflammatory back pain, and extra-articular manifestations such as psoriasis, uveitis associated with IBD, significant familial clustering, and the genetic association with human leukocyte antigen B27 (HLA-B27) (1, 2).

The Assessment of SpondyloArthritis International Society (ASAS) classification criteria for SpA (3–5) include MRI and conventional radiology findings, HLA-B27 testing, and a wider range of clinical features compared with previously available criteria. This allows the coverage of the whole disease spectrum, including mild and early-stage SpA, and distinguishes axial from peripheral disease, with potential implications for therapeutic approaches (6). Sacroiliitis and spondylitis are the hallmarks of axial SpA, while enthesitis and dactylitis are the ones identified for peripheral SpA. However, most patients with axial SpA also present peripheral manifestations. A recent study has demonstrated that AS patients, with or without psoriasis, are demographically, genetically, clinically, and radiographically different from axial PsA patients. Thus, the existing criteria for axial SpA may not encompass its diverse expression (7, 8).

Since 2000, biologic tumor necrosis factor (TNF)-α inhibitors have substantially changed the treatment of SpA. More recently, a better understanding of SpA pathogenesis has led to the identification of newer biologics targeting other inflammatory mediators, e.g., interleukin (IL)-23/IL-17 axis inhibitors, phosphodiesterase 4 (PDE4) inhibitors, and janus kinase (JAK) inhibitors (9).

In this scenario, new epidemiological data on the clinical manifestation and management of this disease and the real-world performance and safety of new agents are necessary (10, 11). Observational registries and large-scale real-world studies are powerful tools for the exploration of these topics and improvement of knowledge in areas not covered by clinical trials (12). Registries in SpA or its subtypes, situated in Europe and the Americas (13, 14), have usually involved patients with longstanding disease and severe disease expression, markedly receiving biologics at enrollment. Patients with mild to moderate SpA or treatment naïve are typically underrepresented.

In Italy, the experience with registries or large clinical databases in rheumatology is limited and includes the following: the Lombardy Rheumatology Network (LORHEN) registry, which is based on patients with rheumatic arthritis (RA) treated with anti-TNF agents since 2009 (15), the Italian Group for the Study of Early Arthritis (GISEA) registry (16), which includes biologics-treated RA and SpA patients since 2003, and a retrospective multicenter study of PsA from the Italian Group for the Study of Psoriatic Arthritis (17). Therefore, data on the clinical and epidemiological profiles of Italian SpA patients, particularly those with an early phase of the disease, are needed.

The Spondyloarthritis Italian Registry: Evidence from a National Pathway (SIRENA) is an Italian registry of SpA patients naïve to any disease-modifying antirheumatic drugs (DMARDs) for SpA. As such, it offers a unique opportunity to explore early SpA in a real-world setting.

In this study, we presented the baseline data of 350 SpA patients enrolled in the above-mentioned SIRENA study to explore the demographic and clinical differences, patient-perceived symptom burden, and physician treatment choices between axial and peripheral SpA and, in each disease group, in men and women.

## Materials and Methods

SIRENA is an ongoing, Italian, observational, prospective study of patients with first or newly confirmed diagnoses of SpA according to ASAS criteria (3–5), and naïve to conventional, biological, and targeted DMARDs for SpA.

This study involved 23 Italian rheumatologic centers. The recruitment started in June 2017 and closed in June 2019. Patients included in the SIRENA study were required to be adults (>18 years), have a first diagnosis or a confirmed diagnosis of SpA according to the ASAS criteria at inclusion, and be naïve to any DMARDs specifically prescribed for SpA. Exclusion criteria were participation in interventional or investigational drug studies in the past 30 days and the inability or refusal to sign the informed consent form, complete patient-reported outcome (PRO) questionnaires, or participate in the 2-year data collection. Participation of the patients in observational non-interventional studies, past or current treatment with biological DMARDs for psoriasis or Crohn's disease, and use of local and/or systemic corticosteroids and non-steroidal anti-inflammatory drugs (NSAID), including coxibs, were allowed.

Patients were consecutively enrolled in the study during consultation visits and then classified as subjects presenting with predominantly axial or peripheral manifestations by the local rheumatologist, who also made a clinical diagnosis of the SpA subtype were also made. After enrollment, follow-up visits were planned throughout the 2-year observation period according to clinical practice, i.e., every 6 months (±2 months) unless clinically indicated otherwise, e.g., in case of flares/consistent therapy modification and physicians check after significant therapy modification. Radiographic and imaging evaluations were not performed by study design but according to routine clinical practice. At each consultation visit, clinicians could access and review previous imaging data available in the medical records of the patients.

The registry collected demographic, lifestyle, and medical data available for clinical practice. These were primarily retrieved from medical records and entered into an electronic case report form (eCRF). Additionally, in accordance with local regulations, participating physicians obtained PRO data. At the baseline visit, the physicians recorded the demographic data, medical history of SpA (including the first specific signs and symptoms of SpA and their time of onset), relevant comorbidities, family history of psoriasis, and selected laboratory values (e.g., rheumatoid factor, HLA-B27, and anti-citrullinated protein antibody) were recorded. In addition, the ClASsification criteria for Psoriatic ARthritis (CASPAR) were evaluated for the PsA subgroup. At each visit, vital signs, lifestyle habits, personal characteristics (smoking, alcohol consumption, body weight, and body mass index [BMI]), physician-evaluated clinical disease manifestations, markers of inflammation (e.g., c-reactive protein [CRP] and erythrocyte sedimentation rate [ESR]), details on SpA therapy(ies) (including any changes in posology and/or drugs), PRO measures, and measures of treatment effectiveness and safety were recorded. SpA treatments were assigned by physicians as per normal clinical practice.

The clinical assessment of SpA included the involvement pattern of the disease (predominant axial or peripheral manifestations), SpA subtypes, number of swollen and tender joints, presence of skin psoriasis [with the body surface area (BSA) affected], nail psoriasis (plus nail count), enthesitis (18) [along with the Maastricht Ankylosing Spondylitis Enthesitis Score (MASES); 0–13], dactylitis (including finger count), fibromyalgia [according to the American College of Rheumatology (ACR) 2010 criteria] (19), the visual analog scale (VAS) for the physician joints, skin, global assessment (PhGA) of the patient, and disease activity. Composite disease activity measures included the Ankylosing Spondylitis Disease Activity Score (ASDAS)-CRP and Bath Ankylosing Spondylitis Metrology Index (BASMI; 0-10) scores for patients with axial SpA, and the Disease Activity Score in 28 joints (DAS28; 0-10) and the Disease Activity index for PSoriatic Arthritis (DAPSA) score for patients with peripheral SpA. In patients with PsA, the state of Minimal Disease Activity (MDA) was evaluated according to Coates et al. (20).

Patient-reported outcome measures were collected through the Bath Ankylosing Spondylitis Functional Index (BASFI; 0–10), Bath Ankylosing Spondylitis Disease Activity Index (BASDAI; 0–10), Health Assessment Questionnaire Disability Index (HAQ-DI; 0–3), Work Productivity and Activity Impairment Questionnaire (WPAI), and patient VAS (0–100) for pain, sleep quality, and global assessment (PtGA). Higher scores reflected worse outcomes for BASFI, BASDAI, HAQ-DI, and VAS scores. The WPAI outcomes were expressed as impairment percentages, with higher numbers indicating greater impairment and less productivity.

The study was approved by the Ethics Committees of the participating centers and conducted according to the principles of the Declaration of Helsinki and the Good Clinical Practice guidelines. All patients provided written informed consent to participate in the study.

In the present report, we described the baseline data of the ongoing prospective SIRENA study; follow-up results will be presented in the future. The Strengthening the Reporting of Observational Studies in Epidemiology (STROBE) guidelines for reporting observational studies were followed (21).

### Statistical Analyses

Data were tabulated separately for axial and peripheral SpA and, in each subgroup, by gender. Descriptive statistics were calculated. Continuous variables were presented as mean ± *SD* or median (range), while categorical variables were presented as absolute numbers and percentages. Differences between axial and peripheral groups in terms of demographic characteristics, clinical features, and treatment patterns were assessed by the chi-square test for categorical variables (for normally distributed variables) or by either the *t*-test or the Mann–Whitney U test (for non-normally distributed variables). All the analyses were performed using the SAS software version 9.4 (SAS Institute, Inc., Cary, NC, USA).

## Results

A total of 350 patients were included in the SIRENA study, with a median age of 50 years old (range: 19–83) at study inclusion; 50.4% of the patients were men. Among the 350 patients, 123 (35.1%) were classified into the axial group and 227 (64.9%) into the peripheral group by clinical assessment.

### Baseline Demographic and Clinical Characteristics

At enrollment, patients with axial SpA were younger than patients with peripheral SpA. There were no significant differences in gender, BMI, smoking habits, alcohol consumption, and family history of psoriasis between the two groups (Table 1). Comorbidities were more frequently reported by patients with predominantly peripheral manifestations (87.2%) than by those with mainly axial disease (62.6%), and, among the latter, more frequently by women (71.9%) than men (53.5%). Cardiometabolic disorders, including hypertension, were reported more frequently by patients with peripheral SpA (33 vs. 18.7% axial), whereas the prevalence of depression was higher in patients with axial SpA (13 vs. 2.6% peripheral). Gastrointestinal diseases were observed in 18.7% of axial and 13.2% of peripheral patients. Eight patients (3.5%) in the peripheral group had malignancies.

**Table 1:** Baseline demographic features and comorbidities in 350 patients with SpA in the SIRENA study by involvement pattern and, in each subgroup, by gender^°^.

	**Axial involvement**			**Peripheral involvement**		
	**All (*n* = 123)**	**Women (*n* = 64)**	**Men (*n* = 58)**	**All (*n* = 227)**	**Women (*n* = 109)**	**Men (*n* = 118)**
Age at inclusion (years), median (min-max)	44 (20–80)	44 (20–80)	44 (22–79)	53^*^ (19–83)	53 (19–83)	52 (22–80)
Men, *n* (%)	58 (47.5)	0 (0)	58 (100)	118 (52.0)	0 (0)	118 (100)
BMI (kg/m^2^), mean (SD)	25.2 (4.8)	24.5 (5.4)	25.9 (3.8)	25.5 (4.3)	24.8 (4.7)	26.1 (3.7)
**BMI categories**^**∧**^, ***n*****(%)**						
Obese	24 (20.3)	12 (19.4)	2 (21.4)	37 (17.4)	17 (17.0)	20 (17.9)
Overweight	23 (19.5)	9 (14.5)	14 (25.0)	47 (22.2)	15 (15.0)	32 (28.6)
Under/normal weight	71 (60.2)	41 (66.1)	30 (53.6)	128 (60.4)	68 (68.0)	60 (53.6)
**Smoking** ^**∧**^						
Never	69 (59.5)	40 (64.5)	29 (53.7)	118 (53.9)	67 (65.1)	51 (44.0)
Former	18 (15.5)	6 (9.7)	12 (22.2)	51 (23.3)	11 (10.9)	40 (34.5)
Current	29 (25.0)	16 (25.8)	13 (24.1)	50 (22.8)	25 (24.3)	25 (21.6)
**Alcohol consumption** ^**∧**^						
Non-drinker	54 (45.8)	37 (57.8)	17 (31.5)	108 (50.2)	66 (65.4)	42 (36.8)
Occasional drinker	60 (50.9)	25 (39.1)	35 (64.8)	92 (42.8)	33 (32.7)	59 (51.8)
Usual drinker	4 (3.4)	2 (3.1)	2 (3.7)	15 (7.0)	2 (2.0)	13 (11.4)
Family history of psoriasis, *n*/tot assessed (%)	36/50 (72.0)	20/29 (69.0)	15/20 (75.0)	92/118 (78.0)	50/61 (82.0)	42/57 (73.7)
Comorbidities, at least one	77 (62.6)	46 (71.9)	31 (53.5)	198^**^ (87.2)	95 (87.2)	103 (87.3)
**Frequent comorbidities**^¥^, ***n*****(%)**						
Cardiometabolic	23 (18.7)	18 (28.1)	5 (8.6)	75^*^ (33.0)	29 (26.6)	46 (39.0)
*Hypertension*	23 (18.7)	15 (23.4)	8 (13.8)	64^*^ (28.2)	28 (25.7)	36 (30.5)
*Dyslipidemia*	19 (15.4)	14 (21.9)	5 (8.6)	29 (12.8)	10 (9.2)	19 (16.1)
*Diabetes*	7 (5.7)	3 (4.7)	4 (6.9)	16 (7.0)	4 (3.7)	12 (10.2)
*MetS*	6 (4.9)	3 (4.7)	3 (5.2)	14 (6.2)	1 (0.9)	13 (11.0)
Gastrointestinal	23 (18.7)	17 (26.6)	6 (10.3)	30 (13.2)	19 (17.4)	11 (9.3)
Endocrine disease	10 (8.1)	6 (9.4)	4 (6.9)	24 (10.6)	19 (17.4)	5 (4.2)
Depression/anxiety	16 (13.0)	13 (20.3)	2 (3.5)	6^**^ (2.6)	6 (5.5)	0 (0)
Osteoporosis	6 (4.9)	3 (4.7)	2 (3.5)	10 (4.4)	6 (5.5)	4 (3.4)
Infections	5 (4.1)	2 (3.1)	3 (5.2)	7 (3.1)	3 (2.8)	4 (3.8)
Malignancies	0 (0)	0 (0)	0 (0)	8^*^ (3.5)	5 (4.6)	3 (2.5)

*BMI, body mass index; MetS, metabolic syndrome; SpA, spondyloarthritis*.

* *p for the comparison between axial and peripheral SpA < 0.05;*

** *p < 0.001*.

° *1 missing data on gender in the axial SpA group*.

∧ *The sum does not add up to the total because of some missing values*.

¥ *Reported by >3% of patients with axial or peripheral SpA; a patient could report one or more comorbidities*.

As for the baseline clinical evaluation (Table 2), median SJC66 and TJC68 were, respectively, 2 and 4 in the peripheral SpA group, while they were both 0 in the axial group. Patients with peripheral SpA had significantly more skin psoriasis (65.2%, mean skin VAS score 17.1 vs. 21.2%, mean skin VAS score 7.4 for the axial group) and nail psoriasis (35.5 vs. 17.1% of the axial group). No significant differences were observed for the presence of dactylitis, enthesitis, and fibromyalgia. In axial SpA, a high level of disease activity was reported according to the ASDAS-CRP (28.8% very high and 50.9% high disease activity). In peripheral SpA, most patients had low-to-moderate disease activity according to DAS28 (45.2% moderate and 16.5% low disease activity) and DAPSA (44.6% moderate and 43% low disease activity). In both SpA groups, women tended to have a higher prevalence of enthesitis and fibromyalgia and higher values of the tender joint count, joint VAS score, and composite scores of disease activity. In the peripheral group, skin psoriasis was somewhat more common in men than in women (70.4 vs. 59.6%), with the former having higher VAS skin (median values 10 vs. 3). In the axial group, nail psoriasis, was more frequent in women (23.8 vs. 7.1%).

**Table 2:** Baseline clinical assessment in 350 patients with SpA in the SIRENA study by involvement pattern and, in each subgroup, by gender°.

	**Axial involvement**			**Peripheral involvement**		
	**All (*n* = 123)**	**Women (*n* = 64)**	**Men (*n* = 58)**	**All (*n* = 227)**	**Women (*n* = 109)**	**Men (*n* = 118)**
CRP (mg/dl), mean (SD) – median	1.1 (1.6) – 0.4	0.9 (1.5) – 0.3	1.2 (1.8) – 0.5	1.0 (1.9) – 0.4	1.0 (2.4) – 0.4	1.0 (1.4) – 0.5
SJC66, mean (SD) – median	3.9 (11.6) – 0	3.7 (10.7) – 0	4.2 (12.9) – 0	2.6 (3.7) – 2.0	2.7 (3.6) – 1.0	2.6 (3.8) – 2.0
TJC68, mean (SD) – median	5.8 (11.6) – 0	7.1 (11.7) – 2.0	4.2 (11.4) - 0	6.8 (8.3) – 4.0	8.7 (9.6) – 6.0	5.0 (6.3) – 3.0
**VAS, mean (SD) (range: 0–100)**						
PhGA score	50.2 (28.6) – 52.0	54.8 (26.7) – 62.0	45.0 (30.1) – 43.5	45.4 (25.9) – 48.5	49.9 (25.6) – 50.0	41.3 (25.6) – 40.0
Joint score	37.6 (32.7) – 30.0	43.2 (32.7) – 50.0	31.1 (32.1) – 20.0	43.4 (36.6) – 40.0	44.9 (25.4) – 48.0	42.0 (44.4) – 40.0
Skin score	7.4 (17.9) – 0	6.6 (16.6) – 0	8.5 (19.5) – 0	17.1** (22.6) – 6.0	14.7 (21.3) – 3.0	19.3 (23.7) – 10.0
Psoriasis skin, *n* (%)	26 (21.2)	13 (20.3)	13 (22.4)	148** (65.2)	65 (59.6)	83 (70.3)
Psoriasis nails, *n*/tot assessed (%)	6/35 (17.1)	5/21 (23.8)	1/14 (7.1)	60/169* (35.5)	26/79 (32.9)	34/90 (37.8)
Number of nails with PsA, mean – median	13.0 – 10.0	13.6 – 10.0	-	6.7 – 4.0	5.9 – 3.0	7.4 – 5.5
Dactylitis, *n*/tot assessed (%)	9/51 (17.6)	6/27 (22.2)	3/23 (13.0)	37/152 (24.3)	12/70 (17.1)	25/82 (30.5)
Number of digits affected, mean – median	8.4 – 12.0	6.7 – 7.0	12.0 – 12.0	1.8 – 1.0	1.9 – 1.0	1.7 – 1.0
Enthesitis, *n*/tot assessed (%)	42/82 (51.2)	26/41 (63.4)	16/41 (39.0)	76/190 (40.0)	45/90 (50.0)	31/100 (31.0)
MASES score, mean – median	4.3 – 2.0	4.2 – 2.5	4.4 – 2.0	3.1 – 2.0	3.3 – 2.0	2.7 – 2.0
Fibromyalgia, *n* (%)	7 (5.7)	6 (9.4)	0 (0)	7 (3.1)	6 (5.6)	1 (0.9)
**In AX SpA**						
ASDAS-CRP, mean (SD)	3.1 (1.3)	3.2 (1.4)	2.8 (1.1)			
**ASDAS-CRP categories**^**∧**^^**¶**^, ***n*****(%)**						
Very high disease activity	31 (28.2)	19 (33.3)	12 (22.6)			
High disease activity	56 (50.9)	29 (50.9)	27 (50.9)	-		
Moderate disease activity	15 (13.6)	5 (8.8)	10 (18.9)			
Inactive disease	8 (7.3)	4 (7.0)	4 (7.6)			
BASMI, mean (SD)	1.9 (1.8)	1.5 (1.5)	2.3 (2.0)			
**In PER SpA**						
DAS28, mean (SD)				3.6 (1.2)	3.9 (1.2)	3.4 (1.2)
**DAS28 categories**^**∧**^^**¥**^, ***n*****(%)**						
High disease activity				25 (13.3)	16 (20.0)	9 (8.7)
Moderate disease activity	-			85 (45.2)	37 (46.3)	48 (46.6)
Low disease activity				31 (16.5)	12 (15.0)	19 (18.5)
Remission				47 (25.0)	15 (18.8)	27 (26.2)
DAPSA, mean (SD)				22.5 (17.1)	26.7 (20.6)	19.0 (12.5)
**DAPSA categories**^**∧**^^**§**^, ***n*****(%)**						
High disease activity				17 (7.8)	10 (11.5)	5 (4.7)
Moderate disease activity	-			86 (44.6)	44 (50.6)	42 (39.6)
Low disease activity				83 (43.0)	31 (35.6)	52 (49.1)
Remission				9 (4.7)	2 (2.3)	7 (6.6)

*ASDAS-CRP, Ankylosing Spondylitis Disease Activity Score with C-reactive protein; CRP, C-reactive protein; DAPSA, disease activity in psoriatic arthritis; DAS28, disease activity score-28; MASES, Maastricht Ankylosing Spondylitis Enthesitis Score; PhGA, physician global assessment; SD, standard deviation; SJC66, swollen joint count in 66 joints; SpA, Spondyloarthritis; TJC68, tender joint count in 68 joints; VAS, visual analog scale*.

* *p for the comparison between axial and peripheral SpA < 0.05;*

** *p < 0.001*.

° *1 missing data on gender in the axial SpA group*.

∧ *The sum does not add up to the total because of some missing values*.

¶ *ASDAS-CRP disease state: < 1.3 inactive, 1.3–2.1 moderate, 2.1–3.5 high, >3.5 very high*.

¥ *DAS28 disease state: < 2.6 remission, 2.6–3.2 low, 3.2–5.1 moderate, >5.1 high*.

§ *DAPSA disease state: < 3.3 low, 3.3–18.5 low, 18.5–45.1 moderate, >45.1 high*.

Except for BASDAI, which showed similar values in the two SpA groups, numerically worse scores in PRO measures were observed among patients with axial SpA (Supplementary Table 1). In both axial and peripheral SpA, PROs were worse among women than men, with the exception of the “% of work time missed” outcome of the WPAI score, which showed higher values in men, at least in the axial group. PtGA values were higher than those of PhGA in each disease group and gender.

### Diagnosis and Time From First Symptoms Onset to Diagnosis

In axial SpA, the most frequent SpA subtypes were AS (*n* = 60, 48.8%), non-radiographic axial SpA (*n* = 24, 19.5%), and undifferentiated SpA (*n* = 19, 15.5%); 17.2% of women with axial SpA had IBD-associated arthritis (Table 3). The large majority of patients with peripheral diseases had PsA (194, 85.5%); the CASPAR criteria for PsA were fulfilled in 94% of these patients. A significantly higher proportion of patients with peripheral than axial SpA had a first diagnosis of SpA at study inclusion (77.1 vs. 58.5%). The pattern of first symptoms differed substantially between the two SpA groups. Almost 80% of patients with axial SpA reported inflammatory back pain as their first symptom; 22% reported to have had arthritis and 11.4% enthesitis (other symptoms were less common). Arthritis (67.8%), psoriasis (skin: 33.9%, nail: 10.6%), enthesitis (24.7% and heel enthesis: 7.1%), and dactylitis (18.5%) were the most common first symptoms reported in patients with peripheral SpA asking for medical consultations. In analyses by gender, women reported arthritis more frequently than men in axial SpA (28.1 vs. 15.5%) and enthesitis in peripheral SpA (34.9 vs. 15.3%). The median time from symptom onset to diagnosis was 36 months in the axial and 24 months in the peripheral group. The rate of HLA-B27 positivity was significantly higher in patients with axial (48%) than peripheral SpA (7.9%).

**Table 3:** Baseline diagnostic features in 350 patients with SpA in the SIRENA study by involvement pattern and, in each subgroup, by gender°.

	**Axial involvement**			**Peripheral involvement**		
	**All (*n* = 123)**	**Women (*n* = 64)**	**Men (*n* = 58)**	**All (*n* = 227)**	**Women (*n* = 109)**	**Men (*n* = 118)**
New diagnosis, *n* (%)	72 (58.5)	42 (65.6)	29 (50.0)	175** (77.1)	82 (75.2)	93 (78.8)
Type of SpA**, *n* (%)						
Ankylosing spondylitis	60 (48.8)	22 (34.4)	38 (65.5)	2 (0.9)	1 (0.9)	1 (0.9)
Non-radiographic axial SpA	24 (19.5)	12 (18.8)	12 (20.7)	1 (0.4)	1 (0.9)	0 (0)
Arthritis with IBD	11 (8.9)	11 (17.2)	0 (0)	12 (5.3)	7 (6.4)	5 (4.2)
Psoriatic arthritis	9 (7.3)	7 (10.9)	2 (3.5)	194 (85.5)	91 (83.5)	103 (87.3)
Undifferentiated SpA	19 (15.5)	12 (18.8)	6 (10.3)	18 (7.9)	9 (8.3)	9 (7.3)
Time from symptoms to diagnosis (months)^¥^, median (range)	36 (3–360)	36 (3–360)	45 (7–240)	24 (1–324)	21 (1–240)	24 (2–324)
SpA first symptoms^¶^, *n* (%)						
Arthritis	27 (22.0)	18 (28.1)	9 (15.5)	154** (67.8)	79 (72.5)	75 (63.6)
Enthesitis	14 (11.4)	9 (14.1)	5 (8.6)	56* (24.7)	38 (34.9)	18 (15.3)
Heel enthesitis	3 (2.4)	2 (3.1)	1 (1.7)	16 (7.1)	8 (7.3)	8 (6.8)
Dactylitis	9 (7.3)	7 (10.9)	2 (3.5)	42* (18.5)	20 (18.4)	22 (18.6)
Inflammatory back pain	98 (79.7)	49 (76.6)	49 (84.5)	40** (17.6)	21 (19.3)	19 (16.1)
Psoriasis skin	12 (9.8)	5 (7.8)	7 (12.1)	77** (33.9)	33 (30.3)	44 (37.3)
Psoriasis nail	2 (1.6)	2 (3.1)	0 (0)	24* (10.6)	12 (11.0)	12 (10.2)
Uveitis	6 (4.9)	4 (6.3)	2 (3.5)	1* (0.4)	1 (0.9)	0 (0)
Crohn disease	3 (2.4)	3 (4.7)	0 (0)	7 (3.1)	3 (2.8)	4 (3.4)
Ulcerative colitis	5 (4.1)	5 (7.8)	0 (0)	2* (0.9)	1 (0.9)	1 (0.9)
Other symptoms	6 (4.9)	2 (3.1)	4 (6.9)	14 (6.2)	7 (6.4)	7 (5.9)
HLA-B27 positive, *n*/tot assessed (%)	36/75 (48.0)	15/37 (40.5)	21/38 (55.3)	3/38** (7.9)	2/22 (9.1)	1/16 (6.3)

*HLA, human leukocyte antigen; IBD, inflammatory bowel disease; SpA, spondyloarthritis*.

* *p for the comparison between axial and peripheral SpA < 0.05;*

** *p < 0.001*.

° *1 missing data on gender in the axial SpA group*.

¥ *Among 81 patients (45 women and 37 men) with axial SpA (65.9%) and 125 patients (62 women and 63 men) with peripheral SpA (55.1%) with diagnosis after ≥1 month from symptom onset*.

¶ *A patient could report one or more symptoms*.

### Treatment Choice

The treatment strategy chosen at baseline differed significantly according to the SpA involvement pattern. At baseline, 77% of patients with axial SpA started a biological DMARD, including 6 patients (5.8%) receiving a combination of biological and conventional DMARDs, and 15.4% started with an NSAID course (Table 4). Among patients with peripheral SpA, ~55% started a conventional DMARD, 20.6% received a biologic agent, and 6.5% had a combination of conventional and biological DMARDs; ~13% of the patients started NSAID therapy.

**Table 4:** Treatment choice at baseline in the SIRENA study by involvement pattern and, in each subgroup, by gender°.

	**Axial involvement**			**Peripheral involvement**		
	**All (*n* = 123)**	**Women (*n* = 64)**	**Men (*n* = 58)**	**All (*n* = 227)**	**Women (*n* = 109)**	**Men (*n* = 118)**
Treatment choice^∧^*, *n* (%)						
NSAIDs	16 (15.4)	7 (12.5)	8 (17.0)	23 (11.6)	4 (4.2)	19 (18.3)
NSAIDs + paracetamol	0 (0.0)	0 (0)	0 (0)	2 (1.0)	2 (2.1)	0 (0)
Biological DMARDs	74 (71.2)	38 (67.9)	36 (76.6)	41 (20.6)	16 (16.8)	25 (24.0)
Biological DMARDs + conventional DMARDs	6 (5.8)	4 (7.1)	2 (4.3)	13 (6.5)	9 (9.5)	4 (3.9)
Conventional DMARDs	7 (6.7)	6 (10.7)	1 (4.3)	110 (55.3)	57 (60.0)	53 (51.0)
Targeted DMARDs	0 (0)	0 (0)	0 (0)	9 (4.5)	6 (6.3)	3 (2.9)
Paracetamol	1 (1.0)	1 (1.8)	0 (0)	1 (0.5)	1 (1.1)	0 (0)

*DMARDs, disease-modifying antirheumatic drugs; NSAIDs, non-steroidal anti-inflammatory drugs*.

* *p for the comparison between axial and peripheral SpA < 0.001*.

° *1 missing data on gender in the axial spondyloarthritis group*.

∧ *The sum does not add up to the total because of some missing values*.

## Discussion

The data described in this report, based on the ongoing real-world SIRENA study, provided a reliable picture of the clinical profile of SpA patients who were naïve to DMARDs for the disease in Italy. Relevant differences were shown between axial and peripheral SpA in terms of comorbidities, disease phenotype, treatment pattern, and the patient's perceived disease burden. In particular, cardiometabolic comorbidities were more prevalent in patients with predominant peripheral manifestations, while depression and anxiety presented more in those with predominant axial SpA. Skin and nail psoriases were also more common and severe in peripheral vs. axial SpA. Additionally, significant differences were observed in the pattern of the first symptoms: inflammatory back pain was reported more frequently as the first symptom by patients with axial SpA, while arthritis, followed by psoriasis and enthesitis, was reported more frequently by patients with peripheral SpA. Furthermore, the study underlined the substantial differences between the therapeutic managements of these two SpA groups, with patients with axial disease being largely treated with biological DMARDs and those with peripheral SpA with conventional DMARDs.

Prospective disease-based registries are excellent tools for characterizing the epidemiology and natural evolution of a disease. These registries are also useful for exploring treatment performance outside of the clinical trial setting. SIRENA is a registry specifically designed for SpA, which collects prospectively and systematically a broad range of information relevant for the disease in a real-world setting, including demographic and lifestyle characteristics, a comprehensive evaluation of the disease burden from clinical and patient perspectives, and treatment details. One strength of this registry is that it involves a number of tertiary rheumatologic sites across the country and gathers data uniformly across centers under strict quality control, even in a real-life setting. Therefore, the SIRENA database is an invaluable source of information on the clinical presentation and management of DMARD-naïve SpA, which has been poorly investigated so far. Indeed, most currently available registries and observational studies in SpA are based on patients with a high burden of disease who are already receiving biologics at study inclusion. In the future, the 2-year follow-up of patients will allow the exploration of the natural evolution of the disease and the effectiveness and safety of the new drugs and combination therapies in an SpA population, typically underrepresented in registrative clinical trials. The database also provides the opportunity to address some topics that have not been extensively investigated in the SpA field, such as pain and PROs, disease-associated depression, and concomitant fibromyalgia.

The results of this study are broadly in line with the available epidemiological and clinical evidence on SpA. However, extreme caution is needed when comparing the present results with those from most of the other registries and observational studies, mainly because of the different patient populations. As stated above, the patients who participated in this study were naïve to any DMARDs for SpA. Especially in peripheral SpA, more than half of the patients had low-to-moderate disease activity as measured by appropriate composite endpoints.

Axial SpA, in particular AS, has been long considered a disease with male predominance (22, 23). The almost equal gender distribution observed in the axial SpA group as a whole in this study derived from a higher proportion of women (vs. men) in the arthritis with IBD, axial PsA, and undifferentiated SpA subtypes, and an equal gender distribution in non-radiographic axial SpA [in line with available literature (23)]. In the AS group, our study also reported a higher proportion of men (63.3% of the patients), with a men:women ratio of 1.7:1. This is in line with the most recent evidence (24), which indicated a decreasing men:women ratio compared to previous reports, from 2:1 to 1.2:1 (22, 25).

A high prevalence of comorbidities in SpA has also been documented, with cardiovascular conditions, osteoporosis, gastrointestinal diseases, and depression being the most common (26–28). In this study, 63% of axial patients and 87% of those with peripheral SpA had at least one comorbidity at study inclusion, with cardiometabolic diseases, gastrointestinal disorders, and depression as the most frequently observed concomitant conditions.

Depression was observed in a significantly higher proportion of patients with axial SpA (13%) compared with peripheral SpA (2.6%), the former having higher disease activity and worse PROs. Anxiety and depression have been frequently reported in axial SpA (29, 30), particularly in women (31) as reported here, and found to be closely related to the serious impairment of spinal mobility and physical function and the consequent deterioration in the quality of life. On the other hand, cardiometabolic diseases, including hypertension, were significantly more frequent in patients with predominantly peripheral manifestations (33%) than in those with mainly axial SpA (19%). Psoriatic arthritis is associated with hypertension, obesity, type 2 diabetes, the metabolic syndrome (MetS), accelerated atherosclerosis, and increased cardiovascular morbidity and mortality (32). Disease-specific and traditional risk factors are likely to explain the atherosclerotic burden in PsA patients. Selected immunological factors involved in PsA, including CRP, TNF-α, interferon gamma (IFN-γ), IL-1, IL-6, IL-23, and Th17, may also play a role (33). A study by Mok et al. (34) compared the prevalence of atherosclerotic risk factors and the MetS in patients with chronic inflammatory arthritis and found that these were more prevalent in patients with PsA than AS.

As expected, patients with peripheral SpA showed a significantly higher burden of skin and nail psoriasis than axial patients. Noteworthy, we observed peripheral manifestations such as arthritis, enthesitis, and dactylitis in a considerable number of patients with axial SpA patients; specifically, dactylitis was present in 17% of these patients. Clinical evidence indicates that 30–50% of patients with axial SpA experience arthritis and/or enthesitis at any time during the course of the disease or even before the detection of the axial disease, while dactylitis is a more rare peripheral manifestation (23).

In this study, we observed a low prevalence of fibromyalgia compared with that reported by Jones et al. (35) in a recently published meta-analysis on axial SpA, for which we have no clear explanation. In any case, it is worth noting that there is a wide variation in the prevalence of fibromyalgia in SpA across studies, which is in part, but not entirely, explained by the different criteria used to diagnose SpA and fibromyalgia.

Patients with axial SpA clearly showed more severe disease activity accompanied by worse PROs compared with those with peripheral SpA. Bearing in mind the caution needed in comparing our results with those from other studies, a recent Dutch cohort of SpA applying the ASAS criteria found higher disease activity (as evaluated by the global assessment of the patient and physician), ASDAS-CRP, and BASDAI in axial as compared with peripheral SpA. Interestingly, these results were consistent in the subgroup of patients naïve to anti-TNF therapy (36). A higher burden of disease in axial SpA/AS compared with PsA was also reported by some other (37, 38), but not all (39, 40), studies.

The comparison between physician and patient global assessment by VAS indicated that patients perceived a slightly greater disease burden than the treating rheumatologists reported, as frequently observed in SpA and other inflammatory rheumatic diseases (38, 41, 42).

Emerging evidence suggests gender-related differences in disease manifestation, clinical burden, and treatment response in SpA (31, 43–45) with women generally showing higher disease activity and worse physical functioning and quality of life, and PsA men having more severe psoriasis. In line with that, in our study, in both of the SpA groups, women tended to show a higher frequency of depression, higher joint tenderness, higher disease activity, and worse PROs compared with men. Enthesitis and fibromyalgia, which have been associated with elevated disease activity and poorer functional status (46, 47), were also somewhat more prevalent in women than men, again in line with previous observations (48). In addition, men with predominant peripheral manifestations had psoriasis more often and more severe skin involvement than their women counterparts.

In our study, the degree of delay between diagnosis (in patients receiving a diagnosis for the first time at study inclusion) and treatment (in patients with a confirmed SpA diagnosis) was greater in the axial than in the peripheral disease. This is consistent with other real-world observations (37, 38). Although previous reports suggested a longer delay in women (49), we found similar results in men and women. Late referrals in axial SpA have been well-documented in the scientific literature and has been mainly attributed to the lack of signs and biomarkers unique to the disease and the difficulty of separating inflammatory back pain associated with axial SpA from other forms of back pain (23), which are relatively common in the general population (50). Peripheral manifestations such as arthritis, enthesitis, dactylitis, and psoriasis are more specific signs, which may be recognized earlier by patients themselves and general physicians. The time from symptom onset to diagnosis observed in axial SpA in this study (median time 36 months, calculated among patients with a diagnosis made ≥1 month from symptom onset) was substantially lower compared with the timespan reported in most of the previous reports (5–14 years on average). A progressive decrease in diagnostic and therapeutic delay in axial SpA has been documented over the last decades (51).

In line with the variable degree of association of HLA-B27 with the different SpA conditions (52), a significantly higher proportion of patients with axial rather than peripheral SpA was positive to HLA-B27. However, this prevalence was relatively low. In particular, it was 48% in the overall axial SpA group, reaching 68% in the AS group (data not shown), which is a low prevalence compared with that observed in Western patients [e.g., between 75 and 90% (53–55)], but a similar prevalence to that observed in studies from selected Mediterranean countries (37, 56, 57), including Italy (52, 58). This is not totally unexpected since, in the Mediterranean area, the strength of the association of HLA-B27 with AS is generally lower than that in Northern Europe, mirroring the gradient of B27 distribution from north to south (52). In a recent study from Argentina, the frequency of HLA-B27 in axial SpA was only 43% (59). As for the peripheral group, HLA-B27 status was available only for 17% (38 out of 227) of the patients, and any interpretation of the 8% prevalence of positivity is speculative.

All patients were naïve to any DMARDs for SpA at study inclusion. In line with most recent guidelines (60–63), the large majority of patients with axial SpA in the SIRENA study were initially prescribed biological DMARDs, while conventional DMARDs were the preferred drugs in peripheral SpA/PsA. However, a considerable proportion of DMARD-naïve peripheral SpA patients received a biological therapy and a small proportion of patients with axial SpA were given conventional DMARDs. As for the former observation, the guidelines from the Group for Research and Assessment of Psoriasis and Psoriatic Arthritis (GRAPPA) and from EULAR allow the prescription of a biologic agent after insufficient response to NSAIDs in patients with PsA when enthesitis or axial signs are predominant (64). It is worth noting that ~14% of patients in our cohort were not yet prescribed any DMARDs.

Despite being tertiary care centers, all the sites participating in the SIRENA registry included SpA patients within their designated early arthritis clinics. This accounts for patients with new-onset and/or suspected disease. In addition, all study centers adopted the same standard criteria, i.e., ASAS criteria, to diagnose and include patients. Thus, selection bias toward more severe cases, though cannot be excluded, should have been minimized. According to the study protocol, patients under DMARDs for SpA were excluded, while past or current treatments with biological DMARDs for other conditions, e.g., IBD, were allowed. In these patients, who could not be strictly defined as “DMARD naïve,” SpA may have obtained benefits from these drugs. In any case, the number of biologics-experienced patients for non-SpA conditions is likely to be small. Among the other limitations, we used the MASES enthesitis score that focused more on tender points in the axial skeleton and may not adequately measure the enthesitis burden in patients with peripheral SpA. However, the tool has been widely implemented in PsA research (18). Finally, we cannot exclude a certain degree of underdiagnosis of dactylitis and nail psoriasis, since patients were recruited exclusively in rheumatologic centers without involving dermatologists.

In conclusion, the present study, based on a well-characterized clinical registry in routine-care settings, provided real-world insights on the clinical features of SpA in Italy. The study has underlined the differences between patients with predominantly axial and peripheral manifestations according to the ASAS criteria, particularly in terms of comorbidities, psoriasis, the pattern of first symptoms, and treatment strategies in a cohort of SpA patients naïve to any SpA-specific DMARDs. The study has also suggested possible gender differences for disease activity, disease burden, and depression. Future data from the prospective SIRENA study are awaited, as they will enhance knowledge on SpA and contribute to improving the diagnosis and the assessment of the disease and to defining the best therapeutic approach.

## Data Availability Statement

The datasets presented in this article are not readily available because of legal/commercial restrictions. Requests to access the datasets should be directed to smarelli@its.jnj.com. Access to anonymized individual participant-level data will not be provided for this study as it meets one or more of the exceptions described on https://yoda.yale.edu/ under “Data Use Agreement - Janssen Pharmaceuticals DUA”.

## Ethics Statement

The studies involving human participants were reviewed and approved by Ethic Committees of the participating centers. The patients/participants provided their written informed consent to participate in this study.

## Author Contributions

All authors listed have made a substantial, direct and intellectual contribution to the work, and approved it for publication.

## Funding

This work was sponsored by the Janssen-Cilag SpA, who provided the financial coverage for the data collection, elaboration, and medical writing activity of the study.

## Sirena Study Group

Gerolamo Bianchi; Gabriella Cardinale; Francesco Ciccia; Lorenzo Dagna; Salvatore De Vita; Salvatore D'Angelo; Ennio Giulio Favalli; Rosario Foti; Franco Franceschini; Armando Gabrielli; Bruno Frediani; Giuseppe Galfo; Roberto Gerli; Rosa Daniela Grembiale; Giuliana Guggino; Ennio Lubrano di Scorpaniello; Antonio Marchesoni; Giuseppe Muccari; Roberto Perricone; Roberta Ramonda; Maurizio Rossini; Raffaele Scarpa; Carlo Selmi.

## Conflict of Interest

ML has received honorary fees for conferences and workshops by Janssen, Abbvie, Novartis, Lilly, Celgene, and Pfizer. CS has received research support from Amgen, Janssen, Novartis, and Pfizer and consulting fees, honoraria, and/or speakers bureau from AbbVie, Amgen, Celgene, Eli-Lilly, Janssen, Novartis, Pfizer, and Sanofi-Regeneron. RR has received honoraria and speaker fees from Novartis, Abbvie, Pfizer, MSD, and Janssen. LD has received consultation honoraria from Abbvie, Amgen, Biogen, Celltrion, GlaxoSmithKline, Novartis, Pfizer, Roche, Sanofi-Genzyme, and SOBI outside of the current work. SD'A has received consulting fees and/or speakers bureau from AbbVie, Biogen, BMS, Celgene, Janssen, Lilly, MSD, Novartis, Pfizer, Sanofi, and UCB. EF has received consulting fees and/or speaking engagements from AbbVie, Bristol-Myers Squibb, Lilly, Merck Sharp and Dohme, Pfizer, Galapagos, Sanofi-Genzyme, and UCB. SM and DF are employees of Janssen-Cilag SpA, Italy. The remaining authors declare that the research was conducted in the absence of any commercial or financial relationships that could be construed as a potential conflict of interest.

## Publisher's Note

All claims expressed in this article are solely those of the authors and do not necessarily represent those of their affiliated organizations, or those of the publisher, the editors and the reviewers. Any product that may be evaluated in this article, or claim that may be made by its manufacturer, is not guaranteed or endorsed by the publisher.
