# Supplementary material for: An Italian Disease-Based Registry of Axial and Peripheral Spondyloarthritis: The SIRENA Study
Source: Front Med (Lausanne). 2021 Sep 22;8:711875. doi: 10.3389/fmed.2021.711875 (PMC8492964; doi:10.3389/fmed.2021.711875)
Supplement: Supplementary file 1 [file Table_1.DOCX]

Supplementary Table 1. Physician Global Assessment (PhGA) and Patient-Reported Outcomes (PROs) at baseline in 350 SpA patients* in the SIRENA study by involvement pattern and, in each subgroup, by gender°.

|  | Axial involvement | | |  | Peripheral involvement | | |
| --- | --- | --- | --- | --- | --- | --- | --- |
|  | All  (n=123) | Women  (n=64) | Men  (n=58) |  | All  (n=227) | Women  (n=109) | Men  (n=118) |
| PhGA, n | 115 | 60 | 54 |  | 222 | 105 | 117 |
| mean (SD) | 50.2 (28.6) | 54.8 (26.7) | 45.0 (30.1) |  | 45.4 (25.9) | 49.9 (25.6) | 41.3 (25.6) |
| median (min, max) | 52.0 (0-100) | 62.0 (0-100) | 43.5 (0-100) |  | 48.5 (0-100) | 50.0 (1.0-100) | 40.0 (0-95.0) |
| PtGA, n | 112 | 59 | 52 |  | 209 | 102 | 107 |
| mean (SD) | 56.4 (27.8) | 61.5 (25.8) | 50.3 (29.2) |  | 50.3 (26.2) | 56.4 (23.1) | 44.5 (27.7) |
| median (min, max) | 63.0 (0-100) | 70.0 (2.0-100) | 50.0 (0-100) |  | 50.0 (0-100) | 58.5 (7.0-100) | 47.0 (0-100) |
| Pain VAS score | 113 | 60 | 52 |  | 207 | 101 | 106 |
| mean (SD) | 56.7 (28.3) | 61.1 (26.6) | 50.6 (29.1) |  | 51.9 (26.8) | 57.4 (25.3) | 46.8 (27.3) |
| median (min, max) | 60.0 (0-100) | 69.5 (2.0-100) | 50.0 (0-100) |  | 53.0 (0-100) | 61.0 (0-100) | 48.5 (0-100) |
| Sleep VAS score, n | 113 | 60 | 52 |  | 211 | 103 | 108 |
| mean (SD) | 55.3 (29.3) | 57.4 (29.5) | 52.3 (29.2) |  | 44.0 (30.1) | 50.4 (29.8) | 37.9 (29.2) |
| median (min, max) | 59.0 (0-100) | 61.5 (0-100) | 53.0 (0-100) |  | 44.0 (0-100) | 53.0 (0-100) | 34.0 (0-100) |
| BASFI, n | 110 | 58 | 51 |  | 133 | 65 | 68 |
| mean (SD) | 4.6 (2.8) | 5.2 (2.6) | 3.9 (2.8) |  | 3.5 (2.6) | 4.0 (2.6) | 3.1 (2.4) |
| median (min, max) | 5.1 (0-9.7) | 5.8 (0-9.4) | 3.6 (0-9.6) |  | 2.9 (0-10.0) | 3.9 (0-10.0) | 2.45 (0-8.9) |
| BASDAI, n | 112 | 59 | 52 |  | 139 | 70 | 69 |
| mean (SD) | 5.2 (2.4) | 5.8 (2.3) | 4.5 (2.3) |  | 5.2 (2.3) | 5.8 (2.1) | 4.6 (2.3) |
| median (min, max) | 5.5 (0-9.3) | 6.2 (0-9.3) | 4.5 (0.3-9.2) |  | 5.5 (0.2-10.0) | 6.1 (1.0-10.0) | 4.8 (0.2-9.2) |
| HAQ-DI score, n | 109 | 58 | 50 |  | 203 | 99 | 104 |
| mean (SD) | 0.9 (0.7) | 1.1 (0.7) | 0.6 (0.6) |  | 0.7 (0.7) | 0.9 (0.7) | 0.6 (0.6) |
| median (min, max) | 0.8 (0.0-2.5) | 1.1 (0-2.5) | 0.5 (0-2.3) |  | 0.6 (0.0-2.8) | 0.8 (0-2.8) | 0.4 (0-2.6) |
| WPAI |  |  |  |  |  |  |  |
| *% work time missed, n* | 49 | 19 | 30 |  | 107 | 45 | 62 |
| mean (SD) | 7.3 (21.4) | 4.2 (9.5) | 9.2 (26.3) |  | 8.8 (24.7) | 8.6 (25.6) | 8.9 (24.3) |
| median (min, max) | 0 (0-100) | 0 (0-35.1) | 0 (0-100) |  | 0 (0-100) | 0 (0-100) | 0 (0-100) |
| *% impairment at work, n* | 67 | 33 | 34 |  | 134 | 61 | 73 |
| mean (SD) | 48.2 (31.9) | 58.5 (26.6) | 38.2 (33.7) |  | 39.7 (31.4) | 45.4 (30.9) | 34.9 (31.2) |
| median (min, max) | 50.0 (0-100) | 60.0 (0-100) | 25.0 (0-100) |  | 40.0 (0-100) | 50.0 (0-100) | 30.0 (0-100) |
| *% overall work impairment*, n | 48 | 19 | 29 |  | 106 | 45 | 61 |
| mean (SD) | 44.1 (33.0) | 52.4 (27.9) | 38.7 (35.3) |  | 40.1 (33.0) | 45.1 (33.1) | 36.4 (32.7) |
| median (min, max) | 45.0 (0-100) | 60.0 (0-100) | 20.0 (0-100) |  | 40.0 (0-100) | 50.0 (0-100) | 30.0 (0-100) |
| *% activity impairment*, n | 100 | 53 | 46 |  | 183 | 93 | 90 |
| mean (SD) | 56.7 (28.6) | 63.4 (23.9) | 48.0 (31.0) |  | 48.5 (30.3) | 55.3 (28.7) | 41.4 (30.4) |
| median (min, max) | 60.0 (0-100) | 70.0 (0-100) | 50.0 (0-100) |  | 50.0 (0-100) | 60.0 (0-100) | 40.0 (0-100) |

Abbreviations: BASDAI: Bath Ankylosing Spondylitis Disease Activity Index; BASFI: Bath Ankylosing Spondylitis functional index; HAQ-DI: Health Assessment Questionnaire Disability Index; PhGA: Physician global assessment of patient; PsA: Psoriasis arthritis; PtGA: Patient’s global assessment; SD: standard deviation: VAS: Visual analogue scale; WPAI: Work Productivity and Activity Impairment.

* The sum does not add up to the total because of some missing values.

° 1 missing data on gender in the axial SpA group.
